# Identifying pediatric extrapulmonary tuberculosis through an active screening program: Findings and programmatic implications from Bangladesh

**DOI:** 10.1371/journal.pgph.0006971

**Published:** 2026-08-19

**Authors:** Giordano Sosa Soto, Jeffrey I. Campbell, Amyn A. Malik, Md. Mahfuzur Rahman, Farzana Hossain, Suchitra Kulkarni, Kamruzzaman Kamrul, Md. Toufiq Rahman, Jacob Creswell, Hamidah Hussain, Tapash Roy, Meredith B. Brooks

**Affiliations:** 1 Department of Pediatrics, Boston Children’s Hospital, Boston, Massachusetts, United States of America; 2 Boston Medical Center, Boston, Massachusetts, United States of America; 3 IRD Global, Singapore; 4 UT Southwestern Medical Center, Dallas, Texas, United States of America; 5 IRD Bangladesh, Dhaka, Bangladesh; 6 Bangladesh Shishu Hospital & Institute, Dhaka, Bangladesh; 7 Innovations & Grants Team, Stop TB Partnership, Geneva, Switzerland; 8 Department of Global Health, Boston University School of Public Health, Boston, Massachusetts, United States of America; University of Sydney, AUSTRALIA

## Abstract

We assessed 188 children diagnosed with extrapulmonary tuberculosis in Bangladesh. Over half were aged 10–14 years old. Common symptoms included fever (91.5%), weight loss (88.3%), and decreased appetite (72.9%). Four children died prior to treatment; of 176 children on treatment, 4.0% had unsuccessful outcomes, potentially influenced by age and low weight. Findings highlight the need for improved diagnosis and management in children.

## Introduction

Extrapulmonary tuberculosis (EPTB) poses significant diagnostic and therapeutic challenges, particularly in children [1]. Invasive procedures for diagnostic purposes may be complex or risky in children, particularly in resource-constrained settings. Furthermore, the paucibacillary nature of EPTB can limit the sensitivity of microbiological tests [2]. Consequently, the diagnosis of pediatric EPTB often relies heavily on clinical judgment and imaging. EPTB represented 16% of notified new or relapsed cases of tuberculosis (TB) in 2023 [1]. Globally, an estimated 1.3 million children and young adolescents (0–14 years) developed TB in 2023, representing approximately 12% of the total TB burden [1]. Among notified pediatric cases, the proportion with extrapulmonary involvement varies widely by setting and definition used. While overall treatment success for notified pediatric TB is high, data disaggregated by extrapulmonary disease and age subgroups are limited, particularly in high-burden settings. In Bangladesh—one of the 30 high TB-burden countries— diagnosis and management of pediatric TB, including EPTB, follow the National TB Program guidelines, which are aligned with WHO recommendations for symptom-based screening and clinical diagnosis in children. [3] Further, only 4% of new TB cases in Bangladesh were <15 years old; 77% of these pediatric cases were reported as having pulmonary TB [1]. Bangladesh provides a valuable context for studying diagnosis and treatment outcomes of children with EPTB in a high-burden setting [4], given that published data on pediatric EPTB in high-burden settings remain limited, particularly regarding real-world programmatic experience. This study aims to contribute observational, programmatic data that may inform practice. We aimed to describe the clinical presentation, diagnostic test results, and treatment outcomes of children diagnosed with EPTB in an active screening program in Bangladesh.

## Materials and methods

The active screening program was implemented as part of the TB REACH Wave 6 project in the Mymensingh division of Bangladesh from November 2018 to September 2021. Participating facilities (79 public and 47 private) were selected to represent a mix of levels of care within the division; while geographically diverse within the region, they may not be nationally representative. Children aged 0–14 years presenting to these facilities were eligible for verbal symptom screening for TB using a standardized symptom-based questionnaire designed to detect general features of TB for identifying children with pulmonary TB or systemic illness. The screening questions were not tailored specifically to detect extrapulmonary TB manifestations. Health workers verbally screened pediatric patients and/or their guardians for cough ≥2 weeks, fever (including duration), night sweats, weight loss/failure to thrive, glandular swelling, and TB contact within two years using an electronic screening tool. Children were considered screen-positive for TB if they met any one of the following criteria: TB contact in the past two years, cough >2 weeks, glandular swelling, or the presence of at least two of the following: prolonged fever, night sweats, or weight loss. Further details on the screening tool, implementation, and overall program outcomes are reported elsewhere [5,6].

Children who screened positive were referred for clinical evaluation by trained physicians. Diagnostic evaluation was conducted according to National TB Program Guidelines and included clinical assessment, imaging (chest X-ray, ultrasound, or CT when available), microbiologic testing (GeneXpert or smear microscopy when possible), and histopathology where feasible. The specific diagnostic approach varied by clinical presentation and resource availability at the facility level. Prior to program initiation, all clinicians involved in pediatric TB diagnosis received training on the National TB Guidelines. Continuous monitoring of diagnosis and treatment was provided with support from national and provincial TB programs, including re-trainings as needed. Children with suspected extrapulmonary TB were referred for further evaluation based on clinical findings suggestive of localized disease (e.g., lymphadenopathy, abdominal findings, or focal symptoms), although no standardized algorithm for EPTB-specific referral was applied uniformly across all sites.

Data included demographics, symptoms, physical exam findings, diagnostics (X-ray, ultrasound, CT, GeneXpert, smears, histopathology), BCG status, contact history, and treatment details. TB was diagnosed by bacteriologic confirmation or clinical criteria. Pulmonary TB was defined as TB with any lung involvement (including intrathoracic disease), consistent with the WHO consultation on the classification of intrathoracic TB disease in children. EPTB was defined as disease involving only extrapulmonary sites; excluding cases with concurrent pulmonary TB involvement, consistent with WHO recommendations. [3] This definition differs from some epidemiologic classifications that include cases with both pulmonary and extrapulmonary involvement as EPTB and may contribute to a lower observed proportion of EPTB in our study.

### Statistical analysis

Outcomes were categorized as successful (cured, completed) or unsuccessful (failure, death, loss to follow-up). Descriptive statistics summarized characteristics, and Chi-squared/Wilcoxon tests compared outcomes; statistical significance was set at p < 0.05. Analyses were conducted in RStudio. Data were accessed for research purposes initially on March 25^th^, 2024. The authors did not have access to information that could identify individual participants during or after data collection.

### Ethical considerations

The study protocol was approved by the Institutional Review Board (IRB) of BRAC James P Grant School of Public Health, BRAC University, Bangladesh (2019–037-ER). Verbal consent was obtained from guardians and children over age seven. Analysis of de-identified data was deemed non–human subjects research by IRBs at Boston University (H-43034) and Boston Children’s Hospital (IRB-P00047239).

## Results

A total of 1,013,746 children were screened through the program. Among them, 20,209 screened positive and were referred for evaluation. The age distribution of those who screened positive was as follows: 25.0% were 0–4 years old, 40.4% were 5–9 years old, and 34.1% were 10–14 years old. Overall, 2,189 children (0.2% of those screened) were diagnosed with tuberculosis. Pulmonary TB accounted for most cases across all age groups, comprising 93.7% of TB cases in children aged 0–4 years, 92.5% in those aged 5–9 years, and 89.6% in the 10–14-year age group. A total of 188 children (8.6% of all diagnosed cases) were diagnosed with EPTB. Of these, over half were male (97, 51.6%), and the most common age group was 10–14 years (100, 53.2%) (Table 1).

**Table 1:** Demographic, clinical, and diagnostic characteristics of all 188 children diagnosed with extrapulmonary tuberculosis (EPTB).

Characteristic	Value / Sub-category	Denominator (N)*	Diagnosed with EPTB
**Sex**	Male	188	97 (51.6%)
**Age**	Median (IQR)	188	10 (6-13)
	0-4 years	188	22 (17.7%)
	5-9 years		60 (31.9%)
	10-14 years		100 (53.2%)
**Weight-for-age percentile**	≤5^th^ percentile	188	93 (49.5%)
**Symptoms**	Cough	184	103 (54.7%)
	Fever	184	172 (91.5%)
	Night sweats	184	91 (48.4%)
	Weight loss	187	166 (88.3%)
	Decreased appetite	184	137 (72.9%)
**Abnormal physical examination**	Chest	184	119 (63.3%)
	Lymph Node	184	90 (47.8%)
	Abdominal	184	62 (32.9%)
**Diagnostic testing suggestive of TB**	Chest Xray	123	63 (51.2%)
	Ultrasound	27	20 (74.1%)
	GeneXpert	5	0 (0%)
	AFB Smear	27	0 (0%)
	Histopathology	73	64 (87.7%)
**Contact with a family member diagnosed with tuberculosis**		188	90 (49.5%)
**Presence of a BCG scar**		188	183 (97.3%)

*The denominator column reflects the number of children with available data for each variable. Where the denominator is 188, the variable was recorded for all children diagnosed with EPTB. Where the denominator is less than 188, this reflects missing data or the subset of children who underwent that specific diagnostic tests. Proportions in the final column are calculated using the relevant denominator for each variable.

Among the 188 children diagnosed with EPTB, the most common presenting symptoms were fever (172, 91.5%), weight loss (166, 88.3%), and decreased appetite (137, 72.9%). Abnormal physical findings were frequent, with abnormal chest examination in 63.3%, lymph node enlargement in 47.8%, and abdominal findings in 32.9% of those with documented exams. Among children who underwent histopathology (73/188, 38.8%) or ultrasound (27/188, 14.4%), results were frequently suggestive of TB: 64/73 (87.7%) for histopathology and 20/27 (74.1%) for ultrasound. These high yields should be interpreted in the context of the selected subgroups who underwent these procedures, likely reflecting high pre-test probability at the time of referral rather than performance across the full cohort. GeneXpert (n = 5) and AFB smears (n = 26) were uniformly negative. A TB contact was reported in 49.5% of cases (Table 1).

Of the 188 children diagnosed with EPTB, four children died before treatment initiation, and eight additional children had no documentation of treatment initiation or follow-up, leaving 176 with treatment outcome data available. Details of the four children who died prior to treatment initiation are shown in Table 2.

**Table 2:** Demographic and clinical characteristics of 4 children with EPTB that died prior to treatment initiation.

Case	Sex	Age (years)	Weight-for-age percentile	Symptoms^a^					Abnormal physical exam^b^			Abnormal diagnostic testing	Outcome
				CGH	FVR	NST	WEL	APL	LN	CST	ABD		
1	F	0	<=3rd	X	X		X			X		Chest x-ray	Died
2	F	3	<=3rd	X			X			X		--	Died
3	M	4	11-25th	X	X		X			X		Chest x-ray, TST	Died
4	F	13	6-10th	X	X		X			X		Chest x-ray, CT scan	Died

a CGH = Cough, FVR = Fever, NST = Night Sweats, WEL = Weight Loss, APL = Appetite Loss

b LN = Lymph node, CST = Chest, ABD = Abdomen

Among the 176 with outcome data, unsuccessful outcomes occurred in 7 (4.0%) children, including 6 (85.7%) who were lost to follow-up and one (14.3%) who experienced treatment failure. When examined by age group, the proportion of children with unsuccessful outcomes was highest among those aged 0–4 years, with 3 out of 19 (15.7%) experiencing an unsuccessful outcome. In comparison, only 1 out of 56 (1.7%) children aged 5–9 years and 3 out of 96 (3.1%) children aged 10–14 years had an unsuccessful outcome (p = 0.02). Children with unsuccessful outcomes more frequently reported weight-for-age < 5^th^ percentile (85.7%) and decreased appetite (100%) compared to children with successful outcomes (48.5% and 75.7%; p = 0.08 and p = 0.07, respectively) (Table 3)

**Table 3:** Characteristics of the 176 children who initiated treatment for extrapulmonary tuberculosis (EPTB), by treatment outcome.

		EPTB treatment initiated (n = 176)	EPTB successful outcome (n = 169)	EPTB unsuccessful outcome (n = 7)	p value (successful vs unsuccessful outcome)
**Sex**	Male	92 (52.3%)	87 (51.5%)	5 (71.4%)	0.78
**Age**	Median (IQR)	10 (7-13)	10 (7-13)	6 (4 – 11)	0.63
	0-4 years	19 (10.8%)	16/19 (84.2%)	3/19 (15.8%)	**0.02**
	5-9 years	56 (31.8%)	55/56 (98.2%)	1/56 (1.8%)	
	10-14 years	96 (54.5%)	93/96 (96.9%)	3/96 (3.1%)	
**Weight-for-age percentile**	<5^th^ percentile	88 (50.0%)	82 (48.5%)	6 (85.7%)	0.08
**Symptoms**	Cough	92 (52.3%)	88 (52.1%)	4 (57.4%)	0.72
	Fever	166 (94.3%)	159 (94.1%)	7 (100%)	0.43
	Night sweats	99 (56.3%)	94 (55.6%)	5 (71.4%)	0.73
	Weight loss	159 (90.3%)	152 (89.9%)	7 (100%)	0.95
	Decreased appetite	135 (70.7%)	128 (75.7%)	7 (100%)	0.07
**Abnormal physical examination**	Chest	111/173 (64.2%)	107/166 (64.5%)	4/7 (57.1%)	0.61
	Lymph Node	86/173 (49.7%)	82/166 (49.4%)	4/7 (57.1%)	
	Abdominal	61/173 (35.3%)	60/166 (36.1%)	1/7 (14.3%)	
**Diagnostic testing suggestive of TB**	Chest Xray	58/117 (49.6%)	55/112 (49.1%)	3/5 (60%)	0.71
	Ultrasound	20/26 (76.9%)	20/26 (76.9%)	--	
	GeneXpert	0/5 (0%)	0/5 (0%)	--	–
	AFB Smear	0/26 (0%)	0/24 (0%)	0/2 (0%)	–
	Histopathology	61/69 (88.4%)	57/65 (87.7%)	4/4 (100%)	0.75
**Time between screening and treatment initiation (days)**	Median (IQR)	7 (3 – 18)	7 (3 – 18)	10 (3.5 – 21.5)	0.63
**Contact with a family member diagnosed with tuberculosis**		88 (50%)	84 (50.6%)	4 (57.1%)	0.98
**Presence of a BCG scar**		172 (97.7%)	165 (97.6%)	7 (100%)	0.89

Note: Successful outcome = cured or treatment completed. Unsuccessful outcome = treatment failure, death during treatment, or loss to follow-up.

## Discussion

Only 4% of notified TB cases in Bangladesh are among children <15 years of age [1], likely an underestimation given that children represent approximately 12% of global incident TB cases [1]. Data on the clinical characteristics and outcomes of pediatric EPTB specifically are limited in high-burden settings, and programmatic real-world evidence is especially scarce. This study aimed to address that gap by reporting findings from a large active screening program, with the understanding that these data are subject to the operational constraints and limitations inherent in programmatic implementation. In our active screening program, TB was diagnosed in only 0.2% of over one million children screened. This low yield reflects the general challenge of detecting TB through broad symptom-based screening in resource-limited settings, and is not specific to EPTB. The specific challenges of diagnosing EPTB in children include reliance on clinical judgment and imaging in the absence of bacteriologic confirmation, which is limited by the paucibacillary nature of EPTB and the difficulties of obtaining samples from extrapulmonary sites in pediatric patients. Importantly, sputum induction and gastric aspiration—while useful for pulmonary TB diagnosis—are not the primary diagnostic tools for EPTB and were not routinely employed in this programmatic context. Of the children diagnosed with tuberculosis, approximately 1 in 12 had EPTB. Interestingly, the proportion of EPTB among children in our study was lower than that reported in other studies in similar settings [7]. This may be due to several factors, including differences in access to diagnostics, earlier detection of children in our study through active case finding, variability in the epidemiological definition of EPTB, or other poorly understood drivers of geographically heterogeneous EPTB epidemiology. The lower proportion of EPTB observed in our study may be partially explained by our strict definition, which excluded cases with concurrent pulmonary involvement, whereas some studies classify such cases as EPTB. Unfortunately, more detailed information about disease site location is not available in our dataset, making a sensitivity analysis using a more broad definition impossible.

Most instances of EPTB occurred among children aged 10–14 year. This likely reflects differences in case detection rather than true epidemiologic variation. Older children may be better able to report localized symptoms and are more likely to undergo diagnostic evaluation, such as imaging or biopsy, compared to younger children. These factors may contribute to detection bias and underdiagnosis of EPTB in younger age groups. This pattern, however, is consistent with known increases in TB risk during adolescence, as reported in the literature. [8] A specific study characterizing children hospitalized with TB in Bangladesh reported that 88% of EPTB instances occurred in the 11–18 age group. [9]

Nearly half of EPTB patients were below the 5th percentile for weight-for-age, with a higher proportion among those with unsuccessful outcomes. This aligns with a Peruvian study showing poorer weight gain in children with treatment failure or death [10]. These findings underscore the potential role of weight monitoring as a valuable indicator for predicting treatment outcomes in children undergoing antituberculosis therapy. Undernutrition is an important risk factor for adverse outcomes in individuals with TB [11], and the WHO recommends nutritional assessments for individuals diagnosed with TB. Our findings point towards the importance of this recommendation for children with EPTB in Bangladesh—both to identify children at risk for unfavorable treatment outcomes and to screen for children who may benefit from nutrition supplementation when they engage in TB care.

Unfavorable outcomes were more common in younger, undernourished children. Young age is linked to severe forms of TB [12], which may confer worse outcomes. Given limited age-disaggregated EPTB outcome data in children, our study highlights young children as a high-risk group and underscores the need for targeted prevention.

Unsuccessful outcomes were primarily driven by LTFU (6 of 7 cases), suggesting challenges in retention in care. Potential contributors in this programmatic context include socioeconomic barriers, caregiver burden, distance to facilities, and clinical improvement leading to early discontinuation of treatment. Although we lack program-specific data on reasons for LTFU, interventions such as active patient tracing, community-based follow-up, and enhanced counseling could help improve retention and treatment completion in similar settings.

Our study has some limitations. Our findings may be subject to an underestimation of the true burden of EPTB due to the design and operational constraints of our screening program. While symptom-based screening was used to identify children at risk for TB, these screening tools were not specifically optimized for detecting extrapulmonary or paucibacillary forms of the disease, which may present with nonspecific or localized symptoms. Our data could not determine the EPTB site, culture status, or resistance, which limits the classification of localized versus disseminated disease. The small number of unfavorable outcomes also limited risk factor analysis. While conducted in a populous, diverse region, findings may not generalize to denser urban areas with differing healthcare access and transmission dynamics. Lastly, our study primarily relied on physical exam findings and radiographic features for diagnosis, with pathology performed only for about one-third of patients diagnosed with EPTB. More invasive sampling procedures to obtain tissue from extrapulmonary sites (such as find needle aspiration or tissue biopsy) are not routinely part of programmatic care. Reliance upon history, exam, and imaging to diagnose EPTB in children is common, and the inability to confirm the diagnosis of EPTB may lead to both under- and over-treatment of children with non-specific presentations. We were unable to classify EPTB by anatomical site (e.g., lymphatic, abdominal, meningeal), which limited our ability to assess relationships between presenting symptoms, physical findings, and specific disease manifestations. Consequently, our estimates likely reflect a conservative approximation of the true EPTB burden in this setting. Developing sensitive and specific non-invasive diagnostics for pediatric TB is critical to reduce clinical uncertainty in settings like ours, where cross-sectional imaging and invasive sampling are not always available. Future prospective studies with standardized EPTB case definitions, systematic anatomical site classification, and microbiologic confirmation would help address the limitations of programmatic data such as ours and further characterize risk factors for poor treatment outcomes in pediatric EPTB.

## Conclusions

Our study describes pediatric EPTB diagnosed through a large-scale active screening program in Bangladesh, offering insights into outcomes achievable in similar high-burden settings. By providing age-disaggregated data, we address a critical gap in understanding pediatric EPTB clinical characteristics and treatment outcomes. The findings highlight the need for improved diagnostics tailored for high-burden settings and the importance of screening for undernutrition in affected children. Younger children were more likely to experience unfavorable outcomes, underscoring the importance of early symptom detection. Further research is needed to identify risk factors for poor treatment outcomes in pediatric EPTB.
